# Changes in the mechanical properties of the respiratory system during the development of interstitial lung edema

**DOI:** 10.1186/1465-9921-9-51

**Published:** 2008-06-12

**Authors:** Raffaele L Dellacà, Emanuela Zannin, Giulio Sancini, Ilaria Rivolta, Biagio E Leone, Antonio Pedotti, Giuseppe Miserocchi

**Affiliations:** 1 TBM Lab, Dipartimento di Bioingegneria, Politecnico di Milano University, Milano, Italy; 2 Department of Experimental Medicine, Universita' di Milano Bicocca, Monza, Italy; 3 Department of Clinical and Preventive Medicine, Università di Milano Bicocca, Monza, Italy; 4 Centro di Medicina dello Sport, Ospedale San Gerardo, Monza, Italy

## Abstract

**Background:**

Pulmonary edema induces changes in airway and lung tissues mechanical properties that can be measured by low-frequency forced oscillation technique (FOT). It is preceded by interstitial edema which is characterized by the accumulation of extravascular fluid in the interstitial space of the air-blood barrier. Our aim was to investigate the impact of the early stages of the development of interstitial edema on the mechanical properties of the respiratory system.

**Methods:**

We studied 17 paralysed and mechanically ventilated closed-chest rats (325–375 g). Total input respiratory system impedance (Zrs) was derived from tracheal flow and pressure signals by applying forced oscillations with frequency components from 0.16 to 18.44 Hz distributed in two forcing signals. In 8 animals interstitial lung edema was induced by intravenous infusion of saline solution (0.75 ml/kg/min) for 4 hours; 9 control animals were studied with the same protocol but without infusion. Zrs was measured at the beginning and every 15 min until the end of the experiment.

**Results:**

In the treated group the lung wet-to-dry weight ratio increased from 4.3 ± 0.72 to 5.23 ± 0.59, with no histological signs of alveolar flooding. Resistance (Rrs) increased in both groups over time, but to a greater extent in the treated group. Reactance (Xrs) did not change in the control group, while it decreased significantly at all frequencies but one in the treated. Significant changes in Rrs and Xrs were observed starting after ~135 min from the beginning of the infusion. By applying a constant phase model to partition airways and tissue mechanical properties, we observed a mild increase in airways resistance in both groups. A greater and significant increase in tissue damping (from 603.5 ± 100.3 to 714.5 ± 81.9 cmH_2_O/L) and elastance (from 4160.2 ± 462.6 to 5018.2 ± 622.5 cmH_2_O/L) was found only in the treated group.

**Conclusion:**

These results suggest that interstitial edema has a small but significant impact on the mechanical features of lung tissues and that these changes begin at very early stages, before the beginning of accumulation of extravascular fluid into the alveoli.

## Background

The functional organisation of the lung extracellular matrix comprises basically two large macromolecular families. The fibrillar components, including collagen I and III and elastic fibers, provide the elasticity of the lung tissue on stretching and de-stretching which is mechanically defined as lung compliance, that is the ratio between the change in lung volume and the corresponding change in transpulmonary pressure. Other macromolecular components, that include hyaluronan (HA) and proteoglycans (PGs), fill the voids among the fibrillar structures and the cells and keep the various structures assembled thanks to multiple linkages, allowing however reciprocal movements of the structures [1]. HA and PGs also play an important role in the control of extravascular lung water. Being highly hydrophilic, they can bind water to form gel-like structures and, furthermore, a peculiar physical property of their hydrated state is the mechanical resistance to compressive forces [1,2]. These molecules are therefore mostly involved in the tissue response to edema formation.

Previous studies evaluated the changes in mechanical properties of the respiratory system after inducing pulmonary congestion[3,4] or severe lung edema [5-9], but no measurements were reported in the condition of interstitial lung edema in vivo implying an increase in extravascular lung water that does not exceed 5–10%.

We wondered whether the mechanical properties of lung tissue are significantly affected when the scaffold of the extravascular matrix is put under tension by interstitial edema. To this purpose, respiratory mechanics was monitored by low frequency forced oscillation technique (FOT) [10,11] in closed-chest mechanically ventilated rats by inducing interstitial lung edema by slow rate saline overload[12].

## Methods

### Animal preparation

We studied 17 Wistar male rats (weight 325–375 g). Animal use and care procedures were approved by the institutional animal care and use committee and complied with guidelines set by the American Physiological Society. The animals were randomly assigned to two groups, one group received saline infusion as described below ('treated', n = 8) and the other one was used as control group (n = 9). The experimental protocol was exactly the same for the two groups excluding the infusion of isotonic saline solution which was performed only in the treated group.

The animals were pre-anesthetized by ether vapours, then anesthetized with an intraperitoneal dose of 50% diluted urethane (1200 mg/kg) and placed in a supine position. A jugular vein was cannulated for drug delivery (both groups) and saline infusion (treated only). Tracheostomy was performed, and a 50-mm plastic cannula (2.5-mm inner diameter) was inserted into the distal trachea. Mechanical ventilation was provided by the same device used for the estimation of mechanical impedance as described below with a tidal volume (V_T_) of 2.5 ml and a frequency of 14 breaths/min. A positive end-expiratory pressure of 1 cmH_2_O was maintained for all the duration of the experiment. Before connecting the animal to the ventilator, paralysis was accomplished by pancuronium bromide (1 mg/kg body wt initial dose, supplemented by 0.33 mg/kg every 40 min).

### Protocol and experimental set-up

Once connected to the mechanical ventilator, the animals were ventilated for four hours with the same ventilator settings. During all this period, only the treated group received infusion of saline solution at a constant rate of 0.75 ml/kg/min.

**Figure 1:**
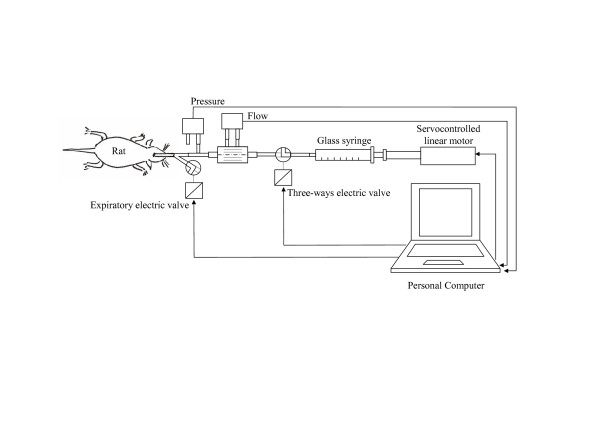
Experimental set-up.

Both mechanical ventilation and the assessment of mechanical impedance were performed by a self-made specially designed mechanical ventilator. The device was obtained by connecting a linear motor (P01-23X80, 44 N peak force, 280 m/s^2 ^max acceleration, Linmot, Spreitenbach, Swiss) provided with its electronic servocontrol unit (E100-AT, Linmot, Spreitenbach, Switzerland) to a 2.5 ml glass syringe (Figure 1). The position of the piston was controlled by an analog signal generated by a digital-to-analog board (DAQCARD 6036-E, National Instruments, Austin, TX) connected to a personal computer. All the parameters of the servocontroller were optimised by standard control science algorithms on a bench model of rat lung (resistance = 105.10 cmH_2_O·s/L, inertance = 0.32 cmH_2_O·s^2^/L, compliance = 0.35 mL/cmH_2_O). The syringe was connected by short and thick silicon tubes (ID 3.5 mm) to a solenoid three-way valve to allow refilling of the syringe with fresh air during expiration. The valve was connected to a T piece. One side of the piece was connected to the tracheal cannula, the other to a two way solenoid valve used to allow passive exhalation during expiration. Both valves were controlled by the same D/A board and personal computer used to control the linear motor. A special software developed in LabView (National Instrument, Austin, TX) allowed the simultaneous control of piston position and valves and the acquisition of the data from the transducers (see below). This software permits the generation of three different flow waveforms. One was a sinusoidal waveform used to provide ventilation during the experiment, the other two were optimal ventilator waveforms, OVW [10], with seven components each chosen to be non-sum non-difference of order three [13]. In order to characterize the spectra of mechanical impedance with a high number of data points and to maintain a low total power of the forcing signal, we repeated the measurement with two different signals, the first with a base frequencies of 0.078 Hz and the second one with a base frequency of 0.313 Hz (Table 1). As the highest frequency of the second OVW was higher than the overall frequency response of our system, we limited the second waveform to the first 6 components. During the application of the OVW the inspiratory three-way valve connected the syringe to the tracheal cannula and the expiratory two-way valve was closed. In this condition, the lungs were expanded at the lowest frequency component of the OVW with an amplitude similar to the one used for ventilation to avoid the development of atelectasis and to maintain the tidal mechanical stretch also during the assessment of mechanical impedance.

**Table 1:** OVW waveforms characteristics.

**Multiplicative factors (NSND3)**	**2**	**5**	**11**	**19**	**31**	**59**	**103**
**Waveform OVW 1**							
Frequency (Hz)	0.16	0.39	0.86	1.48	2.42	4.61	8.05
Amplitude (a.u.)	0.5	0.18	0.18	0.18	0.18	0.18	0.18
Phase (radiants)	4.95	3.82	4.37	3.67	4.05	4.13	4.02
**Waveform OVW 2**							
Frequency (Hz)	0.62	1.56	3.44	5.94	9.69	18.44	-
Amplitude (a.u.)	0.5	0.18	0.18	0.18	0.18	0.18	-
Phase (radiants)	4.95	3.82	4.37	3.67	4.05	4.13	-

Frequency components, amplitude and phase of the two OVW waveforms used to estimate Zin spectra. Amplitudes are expressed in arbitrary units (a.u.).

The assessment of mechanical impedance required one minute for each waveform with a two min ventilation period between the two OVW. The measurements were repeated every 15 min throughout all the experiment. Since the measurement procedure may induce alveolar derecruitment, after each measurement the lung was inflated to 20–25 cmH_2_O for a few seconds before resuming the baseline ventilation.

At the end of the experiment, the animals were sacrificed by an overdose of urethane. The trachea was tied in the neck, the chest was widely opened and the lungs were excised. One lung was removed after tying its main bronchus in order to prevent collapse, immersed in formaline and subsequently processed using routine histological techniques. The other lung was cut into three-four pieces that were used to compute the wet to dry weight ratio (W/D) as an index of lung water content.

Several slices of the processed lung of each animal were analysed by light microscopy at a magnification of 100×. A pathologist was asked to blindly give a score from 0 to 3 to the degree of perivascular edema, peribronchial edema and alveolar flooding of each animal.

### Measurements

Pressure and flow at the airway opening (Pao and $\dot{V}ao$) were measured by a transducer (PXLA0025DN, Sensym, Milpitas, CA) connected to the tracheal tube and by a Fleisch-type pneumotachograph (model 0000 connected to a pressure transducer PXLA02X5DN, 0–2.5 cm H_2_O; Sensym, Milpitas, CA). All connections were made by thick silicon tubing kept as short as possible to provide a satisfying common mode rejection ratio and frequency response. All the signals were sampled at 200 Hz by the same A/D-D/A board used to control the ventilator and recorded by a personal computer. The flow signal was integrated to give lung volume (V_L_). The volume drift resulted from the integration of the flow signal was removed by estimating the linear trend on the integrated signal and removing it from the recording.

The frequency response of the measuring systems was assessed by using the same mechanical model used to optimize the controller parameters and it was flat up to 20 Hz.

### Data analysis

Estimation of input impedance:

The respiratory system input impedance (Zrs) is defined as the complex ratio between the Fourier transform of the pressure and flow signals measured at the airways opening. It is composed of a real part, called resistance (Rrs), and an imaginary part, called reactance (Xrs), which describes the compliance and inertance properties of the system.

$$Zrs(f)=\frac{Pao(f)}{\dot{V}ao(f)}=Rrs(f)+jXrs(f)$$

where f is frequency and j is the imaginary unit.

Pao and $\dot{V}ao$ signals were low-pass filtered with a cut-off frequency of 20 Hz when the base frequency was 0.07825 Hz and of 40 Hz when it was 0.313 Hz. The signals were resampled at 40 Hz and 160 Hz respectively as in [14]. Zrs was computed as the ratio between the auto-spectrum of the flow signal and the cross-spectrum between the pressure and flow signals, in accordance with the cross-spectrum method [15]. The spectra were obtained by averaging ~5 periodograms computed by applying the Fast Fourier Transform to data segments of 512 samples each. The coherence functions of the impedances were computed as described in [16]. In the present study we considered only impedance data with a coherence value greater than 0.95. Data at the oscillatory frequency of 4.61 Hz were discarded as in several measurements the coherence was lower than the threshold likely because of the effect of cardiac activity.

### Model fitting

An empirical model of the respiratory impedance was fitted on Zrs data using the least square method, in order to partition airways and tissue mechanics. The model comprised an airway compartment with a frequency-independent resistance (Raw) and inertance (Iaw) in series with a constant-phase tissue compartment[17] characterized by tissue resistance or damping (G) and elastance (H) according to the following equation:

$$\text{Zrs}=\text{Raw}+\text{j}\omega \text{Iaw}+\frac{\text{G-jH}}{\omega^{\alpha }}$$

Where $\alpha =\frac{2}{\pi }\arctan(\text{H}/\text{G})$ and *ω *= 2*π*f.

Raw was corrected for the contribution of the tracheal tube.

Tissue hysteresivity (*η*) was calculated as the ratio G/H[18].

All the data are expressed as mean ± SD. Significance of differences was tested by two-way ANOVA for repeated measurements using time and treatment group as factors. Multiple comparison after ANOVA was performed using Holm-Sidak test with control group and baseline as control conditions. Statistical tests were performed with a significance level of p < 0.05.

## Results

The interstitial edema induced by saline infusion caused a significant increase in the W/D ratio and in the histological evaluation of perivascular edema for the treated group (Table 2). No fluid was found in the alveolar spaces, confirming that after four hours of infusion only mild interstitial edema was induced.

**Table 2:** Evaluation of the degree of edema. Wet to dry (W/D) ratio and degree of perivascular edema, peribronchial edema and alveolar flooding scored by light microscopy averaged for control and treated groups. Data are reported as mean ± SD in adimensional unit.

	**Controls**	**Treated**
W/D	4.30 ± 0.72	5.23 ± 0.59
Degree of perivascular edema	0	1.20 ± 0.45
Degree of peribronchial edema	0	0
Alveolar flooding	0	0

**Figure 2:**
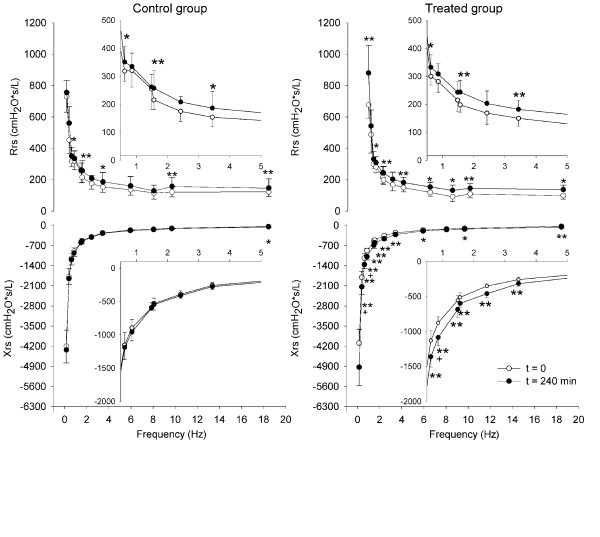
**Effect of interstitial edema on total respiratory system input impedance**. Average ± SD resistance (Rrs) and reactance (Xrs) vs. frequency in the control (left) and treated (right) groups at baseline (open circles) and after 240 min at end-experiment (closed circles). The insets are the enlargement of the graphs in the rage of frequencies between 0.5 and 5 Hz. *: significance of the differences between baseline and end-experiment (* for p < 0.05, ** for p < 0.01); +: significance of the differences between the control and treated group.

The average spectra of Zrs obtained from the control and the treated groups measured at the beginning and at the end of the experiment are reported in Figure 2. For frequencies around 4–5 Hz the coherence was not satisfactory in several tests, likely due to the cardiac artifact. For this reason we decided to exclude the values of Zrs measured at 4.61 Hz from the study. At baseline, there are no significant differences in Rrs and Xrs between the two groups at all frequencies. At the end of the experiment, both the control and treated groups show an increased Rrs. However, the treated group shows a significant increase of Rrs at more frequencies than the controls and the level of significance is greater (p < 0.01) for five frequencies. The reactance spectra shows no significant differences between baseline and end-experiment in the control group. Conversely, in the treated group we found a significant decrease at all frequencies but 8.05 Hz, with p < 0.01 for most of the data points. As the coefficient of variation was similar for both Rrs and Xrs, Xrs is more sensitive than Rrs to the development of interstitial edema.

Because of the wide inter-individual variability of baseline values, there are very slight differences in Zrs at the end of the experiment between control and treated group.

**Figure 3:**
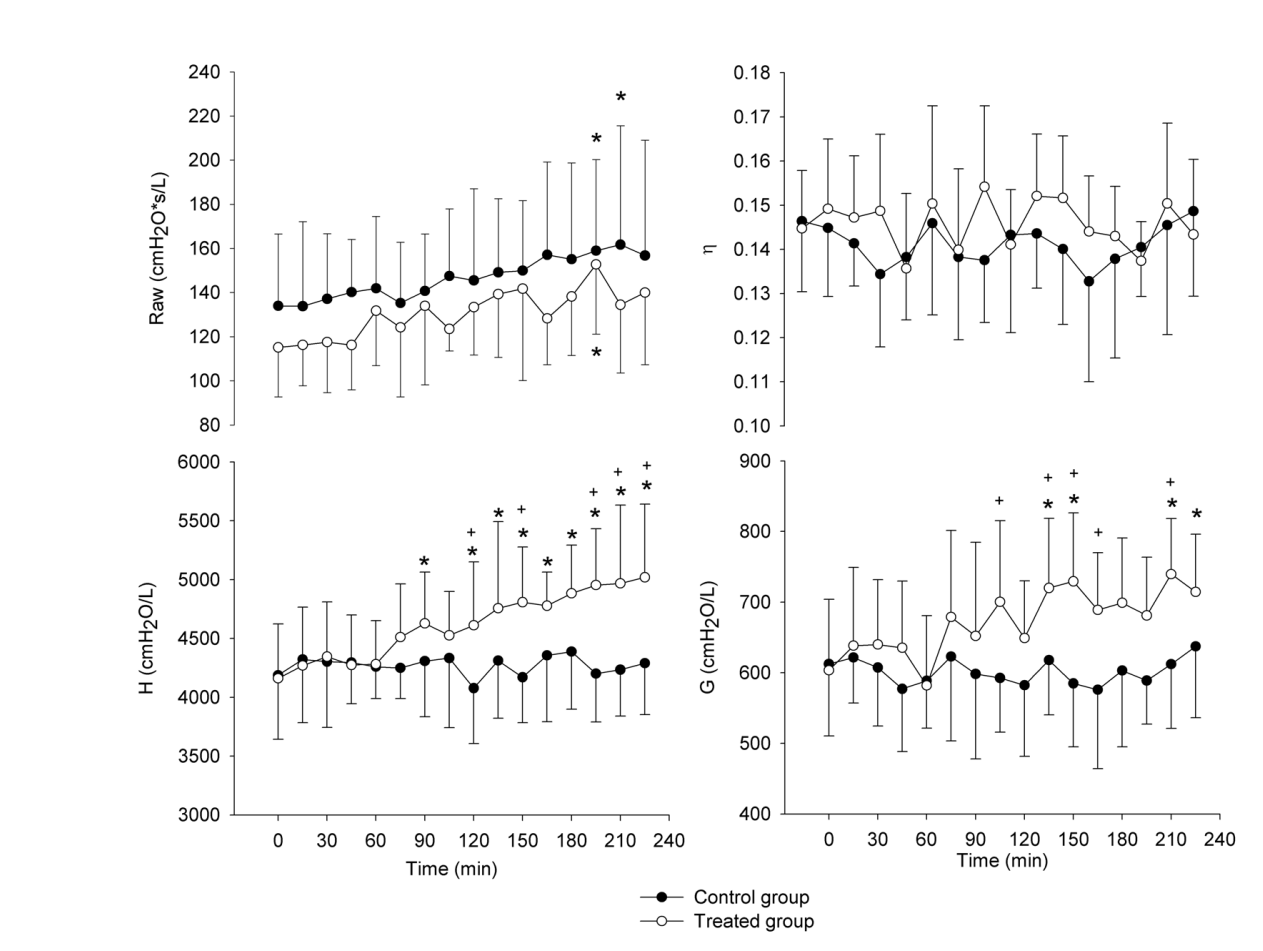
**Changes in Raw and in the constant phase model parameters with time**. Time course of Raw and in the parameters of the constant phase model for the control (closed circles) and treated (open circles) animals (mean ± SD). *: significance of the differences with respect to baseline; +: significance of the differences between the control and treated group.

Figure 3 shows the time courses of Raw and of the estimates of the constant phase model parameters. Raw significantly increases in both the control and the treated group with no significant differences between the two. H increases linearly with time in the treated group, with a highly significant difference from baseline starting from 90 min. However, significant differences relative to control group only appear from 195 min. In the treated group, G increases significantly from baseline values after 135 min, however it shows only a few significant differences compared to the control group. Hysteresivity (*η*) does not reveal a definite trend.

## Discussion

In the present study we investigated the impact of the development of interstitial lung edema on respiratory system mechanical properties. We used low-frequency FOT combined to the constant-phase modeling as this method permits to identify how an experimental intervention differentially affects airway caliber, de-recruitment of lung units and regional heterogeneity of function, and this information cannot be obtained by simpler measures of lung function[19].

The scorings of Table 2 essentially indicate that there is no water accumulation in the alveolar spaces, in agreement with the results that Conforti et al. obtained with a similar model of mild pulmonary edema[20]. Therefore, this study provides the first data concerning modifications in the overall mechanical properties of the respiratory system in vivo during interstitial lung edema measured by low-frequency FOT in absence of airway and alveolar flooding.

### Methodological issues

Anaesthesia and paralysis, together with prolonged mechanical ventilation, can induce changes in lung mechanics per se [21,22], which are likely to be the reason of the slight increase of Raw and G observed in the control group within the four hours. Derecruitment of lung units was prevented by regular positive pressure inflations (recruitment maneuvers) and the use of PEEP, and this was confirmed by the absence of elevation of H with time in the control group.

In our study we used a forcing waveform with different amplitudes for the different frequency components, as the lowest frequency was used to provide also ventilation to the animal. It has been shown that the impedance of the lung is amplitude-dependent [23]. However, this phenomenon was small in the healthy lung and increased markedly after oleic acid injury, where the damage is much more severe than the one induced by our model [6]. The use of two different waveforms allowed us to evaluate the impact of this effect, as the high amplitude frequency in the OVW2 signal used to ventilate the animal is very close to the third small amplitude frequency component of OVW1 (Table 1). As the values of Zrs at these two frequencies are in very good agreement (Figure 2), the use of a higher amplitude at low frequency did not significantly affect the assessment of Zrs in our experimental conditions.

Non-linearity of the respiratory system may also affect the measurements, since the OVW is a composite signal, but the use of appropriate frequency components [13] should have minimized this issue. Moreover, even if this may have affected the absolute values of the impedances reported in figure 2, the possible impact is similar for all the measurements.

Comparing our data with those from other studies, we found that our measurements of Zrs and constant phase model parameters at baseline are similar to those reported in the literature [24]. However, we found slightly lower hysteresivity (*η *= 0.14) compared to [24] (*η *= 0.18) and to [25] (*η *= 0.2). Sakai et al. found that the mechanism that plays a major role in the differences in *η *is heterogeneous constriction of the airways. The lower values of *η *reported in the present paper may be due to the use of OVW, which applies physiological tidal volume amplitudes making the lung more homogeneous than during measurement performed with small amplitude signals [25].

Finally, in this study we are measuring the total respiratory system impedance, which is the result of both lung and chest wall mechanical properties. In particular, the latter constitutes a significant component of the total input impedance [24,26]. However, it has been shown that even during severe edema induced by oleic acid infusion, the chest wall mechanical properties are not affected[6]. These experimental data support the hypothesis that saline infusion does not induce significant changes in the configuration or mechanical properties of the chest wall because, although edema is a general phenomenon affecting all body tissues, the interstitial pressure increases much more in the lung compared to muscles [12] and, accordingly, one would expect the impact of interstitial edema to be much more relevant in the lung compared to the intercostal muscles.

In conclusion, the differences of Zrs between the treated and the control groups and between the baseline and end-experiment in the treated group, shown in figures 2 and 3, should not be affected by all these issues, but they likely reflect the changes in visco-elastic properties of the lung tissue in the specific mechanical conditions of interstitial edema.

### Changes in respiratory mechanics

The accumulation of water in the interstitial space induces significant changes in both Rrs and Xrs and, consequently, also in Raw, G, and H.

Figure 2 shows that in the treated group Rrs increased at all frequencies, while Xrs becomes more negative with a greater decrease at low frequencies. We found that Xrs, which mainly reflects the elastic properties of the tissues or the presence of closure or choke points in peripheral airways [27,28], is much more sensitive than Rrs to the development of interstitial lung edema, displaying significant differences between baseline and end-experiment and between the control and the infused group at more frequencies.

We did not observe an evident transient increase of resistance as reported by Ishii and coworkers [7] which was attributed to vagal reflex. These differences are likely due to the differences in edema model: Ishii et al. induced lung edema by a marked elevation of left atrial pressure, inducing alveolar flooding in less than 100 min. Conversely, our model of lung edema was not associated to significant changes of systemic and lung capillary pressures [12,29].

The differences in Zrs relative to baseline reached statistical significance after 135 min at most frequencies, suggesting that the mechanical properties of the lung are affected during the development of interstitial edema and/or vascular congestion even when the increase of extravascular water is not exceeding 5–10% [12,20].

In order to partition airways and tissue contribution to changes in respiratory mechanics, we fitted the constant phase model on Rrs and Xrs data (Figure 3). Raw slightly increase in a similar way in the control and in the treated group. An increase in Raw can be associated to a reduction of airway calibre but, since in interstitial lung edema we did not find histological evidence of geometrical remodelling of the airways, this small change is likely related to the effects of the prolonged sedation and mechanical ventilation.

In the treated group G and H increased with time. An increase in G and H may be due to increased tissue damping and elastance, reduced lung volume, airway closures or heterogeneous airway constriction. We can exclude heterogeneous airways constriction because it is associated to increased *η *[30], which was not observed in our data. A reduction in lung volume could produce an increase in both G and H, however, *η *is also affected by changes in lung volume [24]. As *η *was constant throughout the experiment, we can conclude that the changes in G and H that we observed are not due to lung volume changes. Finally, also airway closures are very unlikely to occur in our experimental conditions, as suggested also by the histological analysis. For all these reasons the increase in G and H is likely related to changes in the tissue mechanical properties.

### Comparison to previous studies on lung edema

Previous studies correlating the development of lung edema with changes of lung mechanics were focused mainly either on the effects of pulmonary congestion only or on the effects of later stages of acute lung edema.

When the perturbation was limited to pulmonary congestion, mechanical properties of the lung were found to be minimally affected in the range of lung volume considered in our study [3,4,9]. The slight changes previously observed (i.e. decrease in compliance and an increase in pulmonary resistance), are, however, in the same direction as the changes reported in the present study.

Conversely, a major impact on lung mechanics was found when later stages of lung edema were induced by either volume loading [5], increase in microvascular permeability [6,31] increase in left atrial pressure [7,8] or aortic occlusion [9]. However, these models caused pulmonary congestion, alveolar fluid accumulation, small airways compression/occlusion, conditions that clearly impact on the overall mechanical properties of the respiratory system including, of course, airways resistance and lung compliance. A further complication in the evaluation of respiratory mechanics during edema development is the repeated use of BAL that may cause ventilation heterogeneities [31].

## Conclusion

Although the lung seems well designed to resist to edema formation, a sustained condition of interstitial edema is a cause of progressive loss of integrity of the interstitial matrix [32]. The obvious question then is: what is the mechanical resistance of the matrix macromolecules to continuously increased stress? Our experimental evidence is that the transition to severe edema is a matter of minutes [32], suggesting that severe edema acutely develops when the damage to the extracellular matrix overcomes a critical threshold. Therefore, the condition of increased interstitial parenchymal stresses represents an unstable equilibrium between tissue repair and severe tissue lesion. The present data suggest that the assessment of the time course of lung mechanics by forced oscillation technique may provide useful information on the accumulation of water into the lung and/or the development of severe pulmonary congestion. For this reason, the approach developed in this study could have a potential impact for the study of the early phase of the development of lung edema and for the evaluation of possible countermeasures in experimental studies. Moreover, further studies should be addressed to evaluate if the use of FOT can represent a non-invasive, potentially clinically useful tool to detect the earliest stages of congestive heart failure, and institute therapy long before the condition becomes life-threatening.

## Competing interests

The authors declare that they have no competing interests.

## Authors' contributions

RLD Contributed in the design of the study, in writing the manuscript, in the design and development of the experimental set-up and the data processing algorithms, EZ contributed in the in the experimental activity performing the impedance measurements, in drafting the manuscript and in the data processing and statistical analysis, GS Contributed in the design of the study protocol, in the experimental activity, with particular attention to animal preparation, and revised the manuscript, IR Participated in the experimental activity and carried out the processing and measurements of the biological samples, BEL Coordinated and carried out the histological analysis, AP Contributed in the design of the study and revised the manuscript, GM Contributed in the design of the study, in writing the manuscript and coordinated the study. All authors read and approved the final manuscript.

## Grants

This work was partially supported by the Italian Space Agency (ASI), DCMC contract.
